# Ethnic differences in disability-free life expectancy and disabled life expectancy in older adults in Chile

**DOI:** 10.1186/s12877-024-04728-5

**Published:** 2024-01-31

**Authors:** Moisés H. Sandoval, Marcela E. Alvear Portaccio, Cecilia Albala

**Affiliations:** 1 Institute of Nutrition and Food Technology (INTA), University of Chile, Santiago, Chile; 2 National Administrative Department of Statistics of Colombia (DANE), Bogotá, D.C., Colombia

**Keywords:** Disability-free life expectancy, Ethnicity, Older adults, Chile

## Abstract

**Background:**

Although about 10% of the Latin American population is indigenous, ethnic differences in disability-free life expectancy (DFLE) and life expectancy with disability (DLE) are unknown.

**Objective:**

To estimate disability-free life expectancy and disabled life expectancy among Mapuche (the largest indigenous group) and non-indigenous older adults aged 60 years or more in Chile.

**Method:**

Disability was measured following a methodology that combines limitations of daily living, cognitive impairment and dependence previously validated in Chile. Finally, the DFLE was estimated using Sullivan’s method combining life tables by ethnicity and disability proportions from the EDES survey designed for the study of ethnic differentials in health and longevity in Chile.

**Results:**

Non-Indigenous people have a higher total and Disability-free life expectancy compared to Mapuche people at all ages. While at age 60 a Mapuche expects to live 18.9 years, of which 9.4 are disability-free, a non-Indigenous expects to live 26.4 years, of which 14 are disability-free. In addition, although the length of life with disability increases with age for both populations, Mapuche who survive to age 80 or 90 expect to live 84% and 91% of their remaining life with disability, higher proportions compared to non-indigenous people (62.9% and 75%, respectively).

**Conclusions:**

This is the first study addressing inequities in DFLE between the Mapuche and non-Indigenous population, reflected in lower total life expectancy, lower DFLE and higher DLE in Mapuche compared to the non-Indigenous population. Our results underscore the need for increased capacity to monitor mortality risks among older people, considering ethnic differences.

## Introduction

Almost all Latin American countries have benefitted from a reduction in mortality level, which translates to important gains in life duration for the population. This, added to the sustained reduction in fertility levels, has contributed to an accelerated process of population aging. For example, while in the 1960s a Latin American newborn expected to live for 48.6 years, currently expected to live 75.8 years [1]. However, this increase in longevity has not benefitted all societies equally. While life expectancy at birth is 78 years for both sexes in Argentina, in Bolivia it is 68.8 years and Haiti it is less than 65 years. In parallel with the increase in life expectancy, the Latin American population has experimented a relative increase in the prevalence of chronic illnesses and disabilities [2, 3], which are related to the change in the population structure and epidemiological profile of the countries.

The increase in life expectancy, chronic diseases and disability has led to focus attention on the association between “long life” and “quality life”. In fact, the life expectancy is commonly used as an indicator of health, but in recent years it has been emphasized that it is important to consider not only the number of years lived, but also the quality of life—healthy life expectancy [4–7].

Disparities in healthy life expectancy are greater than those detected in life expectancy, given the combination of inequalities in morbidity and mortality [8]. Thus, it is common to find important differences in healthy life expectancy between different population subgroups, which usually indicate health disparities, which, in turn, have their origin in structural inequalities that may have a more pronounced effect on the ethnic minorities. Furthermore, the epidemiological changes that have occurred because of population aging (e.g., increase in chronic diseases) in the different Latin American societies reflect the need to understand ethnic differences not only in the number of years, but also in the quality of the remaining years. In fact, the future long-term care needs of the elderly will be determined by the increase in longevity and whether that increase is accompanied by an expansion or compression of disability at advanced ages [7, 8]. Fundamental information for the design of health strategies that incorporate an intercultural perspective.

Studies in single countries, Argentina [9], Brazil [10–15], Chile [16–20], Colombia [21], México [22, 23], and comparing among Latin American countries and/or cities [24–30] using several health measures (self-reported health, diabetes, depression, disabilities, functional limitations, cognitive deterioration, among others) have shown differences in healthy life expectancy between sexes, ages, socioeconomic status, and residential area. However, even though about 10% of the Latin American population is Indigenous, little is known about ethnic differences in mortality, specifically in relation to the number of years of healthy life or disability-free Indigenous persons can expect to live, compared to the non-Indigenous population. This is complicated because previous studies have shown that indigenous peoples have a higher risk of mortality at all ages [31–34]. Among the reasons for the lack of studies that estimate ethnic differentials in longevity and mortality are: first, the exclusion and structural discrimination of indigenous peoples in Latin American societies, translated into the lack of recognition and autonomy of indigenous peoples. In fact, in Chile the issue of ethnic self-recognition is not considered in vital statistics [35]. Further, there is a lack of reliable and representative information on indigenous populations in nationally representative surveys [31].

In this context is very important to advance to estimate differences in healthy life expectancy by ethnic origin. Understanding that indicators such as healthy life expectancy (in this case, disability-free) that summarize mortality and morbidity are an important tool to recognize how health status and length of life change in populations, allowing us to observe the existing gaps between population subgroups. We believe that our work in Chile can contribute to filling the gap that exists in the region in terms of the study of differences in longevity and mortality between indigenous and non-indigenous older adults. Furthermore, it can encourage addressing this issue in other Latin American societies.

## Chilean context

The Chilean population has benefited from important reduction in mortality and increase in life expectancy. For example, the life expectancy for both sexes increased from 72.6 to 81.2 years in 1990–2023 [1]. In fact, Chileans currently have the longest life expectancy at birth for both sexes in Latin America, followed by Costa Rica (80.3) [1]. However, this increase has not been homogeneous in the subgroups of the population. For example, those with higher socioeconomic status have higher expectancy than the poorest [36, 37]. There is evidence that suggests that Indigenous in Chile have higher mortality as child and as adults [33, 38–41]; results of our previous study describe that the Indigenous have life expectancy at birth 7 years less than non-Indigenous people (76.2 vs. 83.2 years) and even that the Mapuche at birth have a life expectancy 2.1 years less than other indigenous groups (74.6 vs. 76.7 years) [31].

The Chilean population currently includes 18.9 million inhabitants, 12% of which are Indigenous (ethnic self-identification). The Chilean state recognizes the existence of 10 Indigenous groups (Aymara, Atacameño or Licanantay, Chango, Colla, Diaguita, Kawésqar, Mapuche, Quechua, Rapa Nui, Yagán); the Mapuche are the largest group. Of the 1.9 million Indigenous people counted in the 2017 census, 89% were Mapuche [42].

**Fig. 1:**
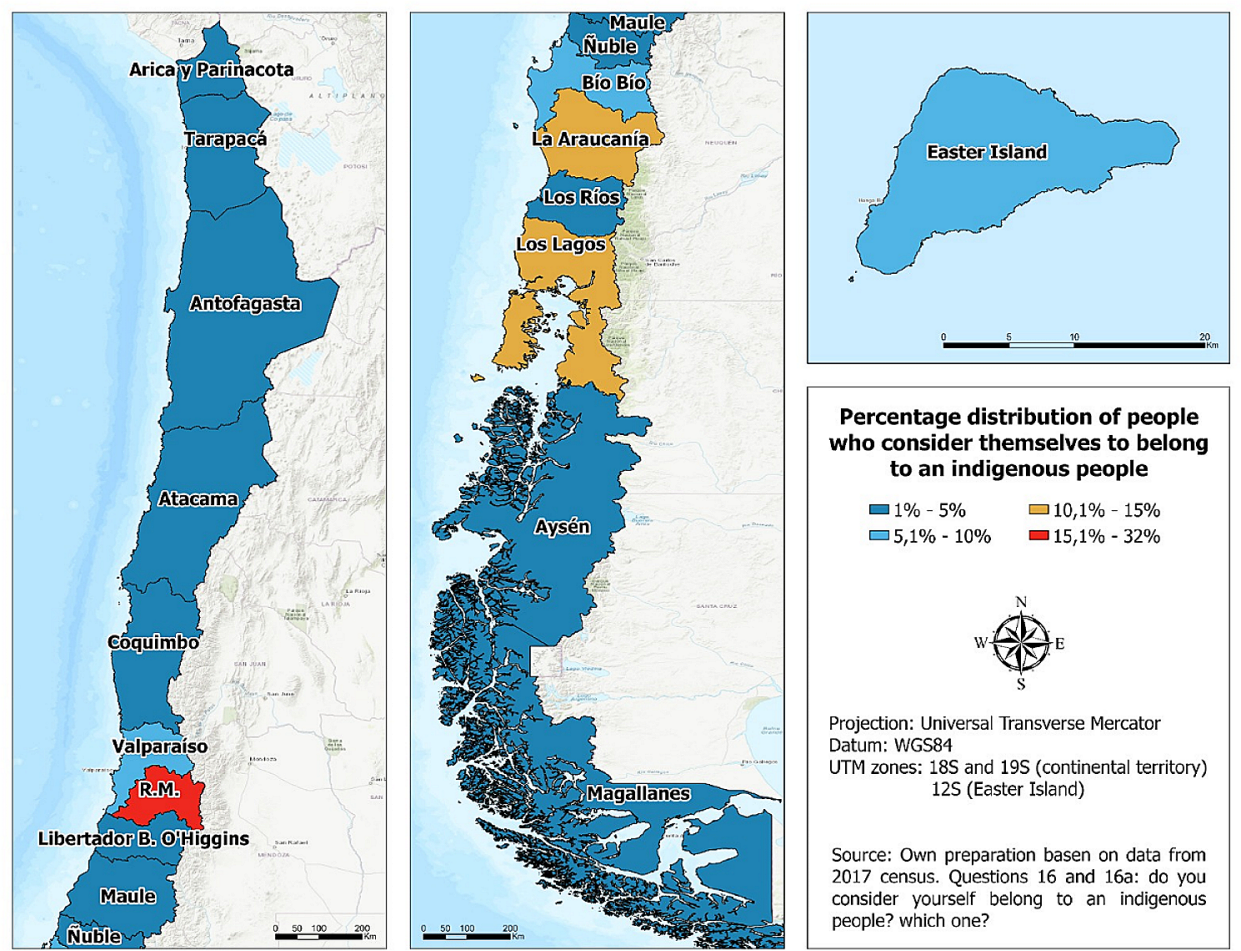
Distribution of the Indigenous population of Chile by region of residence. *Source*: Authors’ elaboration based on the 2017 Census

Most (88%) of the Chilean population lives in urban areas; for Indigenous people it is 80.6%. This urban concentration of Indigenous is due, in large part, to the territorial reductions to which they were submitted by the Chilean state beginning in the XIX century, plus the dynamics of the agrarian crisis that produced a strong migratory process from farms to the cities [43–47]. Of the country’s 16 regions, most of the Indigenous population is in the Metropolitan and La Araucanía Regions (31.8% and 14.7%, respectively [42]); Fig. 1 shows more details.

Indigenous people in Chile and the rest of Latin America are socioeconomically disadvantaged; they have higher poverty and indigence levels than non-Indigenous people [48–50]. The disability-free life expectancy has been estimated for the total Chilean population and for older adults by sex, age, and socioeconomic status [16, 17, 19, 20]. However, no study has examined possible ethnic differences in disability and disability-free life expectancy. Thus, using the life tables by ethnicity for Chile previously published [31], we estimated the disability-free life expectancy and disabled life expectancy of Mapuche and non-Indigenous older adults at 60 years in Chile. We hypothesize that older Mapuche adults have a double disadvantage; lower life expectancy and lower disability-free life expectancy compared to non-Indigenous people.

## Method

### Data

We used data from two sources to estimate the disability and disability-free life expectancy of older Mapuche and non-Indigenous Chileans. First, we used the life tables by ethnicity for both sexes that we previously published [31]. These were elaborated using information from the last Chilean census of 2017 through the indirect demographic method of own children of Brass [51] and we include a differentiated analysis between Mapuche and other Indigenous groups. Secondly, to obtain the prevalence of disability by ethnic origin, we used the Aging, Demography, Ethnicity, and Health EDES (Envejecimiento, Demografía, Etnicidad y Salud in Spanish). The EDES is a survey designed to study ethnic disparities of older adults in Chile. It was applied in 2022 to a probabilistic, geographically stratified, and multi-stage sample, with proportional distribution of the Mapuche and non-Indigenous population of older adults in the Metropolitan and La Araucanía Regions.

A total of 831 persons aged 60 years or more of the rural and urban areas of these regions were sampled with the EDES, as part of the project “Study of the ethnic disparities in health of older adults in Chile”, which was approved by the Scientific Ethnics Committee of the Instituto de Nutrición y Tecnología de los Alimentos (INTA) of the Universidad de Chile.

Finaly, by elderly person in this study we refer to all persons of both sexes who are 60 years of age and older.

#### Disability-free variable

Disability was defined as a non-healthy state of Chilean older adults, evaluated using the methodology suggested by Fuentes et al. [52], the which includes information from the EDES Survey referring to “functional limitations” (basic and instrumental activities of daily living), “cognitive impairment” (Mini mental State Examination) and “dependence” (Pfeffer scale). Therefore, an older person without disability is defined as a person who has no problems in performing basic activities of daily life (ADL) (e.g. dressing, bathing, eating, toilet use, getting in and out of bed, walking) and with no difficulty or only one difficulty in instrumental activities of daily life (IADL) (e.g. shopping, doing housework, using the phone and looking up numbers, managing finances, managing medications). A disability state refers to having limitation in at least one ADL, in two or more IADL or a has score less than 21 on the Mini Mental State Examination (MMSE), previously validated in Chile [53] and greater than 5 points on the Pfeffer Functional Activities Questionnaire (PFAQ). In summary, disability-free life expectancy is defined here as that number of years that older adults can expect to live without difficulties in performing ADL and IADL, and with no cognitive deterioration or dependence.

#### Sullivan method

We used the method of Sullivan [54] to estimate disability and disability-free life expectancy. Its advantages include the fact that it allows using transversal data, it is easy to apply, and its results are relatively sure^47^. Life expectancy calculated by Sullivan’s method gives the number of remaining years that a person of a given age may expect to live in each health state. Sullivan’s method generally reflects the current health of a population, adjusted for mortality levels [55].

The traditional formula to estimate disability-free life expectancy is the following:

$$DFL{E_{\bf{x}}}\, = \,{{\sum {\left( {_n\,{\pi _x}} \right){\,_n}L{\,_x}} } \over {{l_x}}}$$

**DFLE**_**x**_: mean number of healthy years (free of disability and with the perception of good health for a person of age *x*.

_**n**_π_**x**_: prevalence of a health condition in an age group *x* to *x + n.*

_**n**_L_**x**_: person-years lived from *x* to *x + n*, which corresponds to the total numbers of years lived by the cohort in the interval;

**l**_**x**_: probability of surviving to age *x*.

The disabled life expectancy was obtained by subtracting the disability-free life expectancy from the total life expectancy.

It is important to note that disability and disability-free life expectancy were calculated for both sexes together, given that there are no Chilean life tables classified by sex and ethnic origin that would make it possible to analyze ethnic differences in longevity by sex. Also, vital statistics such as death certificates in Chile do not include ethnic origin [35], which impedes the calculation of life tables by ethnic origin and sex.

## Results

Table 1 shows the main characteristics of Indigenous and non-Indigenous Chilean older adults. Non-Indigenous older adults have seven years greater life expectancy at age 60 than Mapuche (26.4 and 18.9 years, respectively). The mean ages of the sampled groups were almost identical; the largest group interviewed was between 60 and 69 years in both cases. Table 1 also shows that older Mapuche have more limitations than their non-Indigenous counterparts. This is true for ADL, IADL, cognitive deterioration and dependence.

**Table 1:** Characteristics of indigenous (Mapuche) and non-Indigenous older adults in Chile

Indicators	Mapuche	Non-Indigenous
Life expectancy at 60y (years)^a^	18.9	26.4
Age (mean, s.d.)^b^	71.4 (8.1)	70.7 (7.9)
60–69 years (%)^b^	48.7	55.4
70–79 years (%)^b^	34.7	26.8
80–89 years (%)^b^	15.1	15.8
90 or more (%)^b^	1.5	2.0
Limitation in one or more ADL (%)^b^	36.5	30.7
Limitation in two or more IADL (%)^b^	43.5	31.4
Mini mental (MMSE) < 21 (%)^b^	7.3	4.4
Pfeffer ^b^ >5	14.9	8.7
Functional limitation (%)^b^	51.4	39.9

*Source*: Own elaboration using EDES Survey and Life Table by Ethnicity [31]

^a^ Sandoval et al., 2023, ^b^ EDES Survey.

Table 2 shows the results of total life expectancy, disability and disability-free life expectancy by age and ethnic origin (Mapuche and non-Indigenous). These results indicate that non-Indigenous persons have a longer total life expectancy and disability-free life expectancy than Mapuche, in all age groups. For example, a 70-year-old Mapuche can expect to live 11 more years, four of which would be disability-free, in contrast to a non-Indigenous person of the same age, who can expect to live 17 more years, eight of them disability-free. Although the ethnic differences in total life expectancy decrease (seven years at age 60 vs. one year at 90+), at age 60 the disability and disability-free life expectancy of Mapuche older adults is almost identical. In fact, the DLE / DFLE ratio accounts for this. However, those Mapuche who manage to survive to age 80 or 90 + can expect to live 5 or 11 times longer with disability, respectively, than they can expect to live healthy (DLE/DFLE ratio). Although as expected this tendency also occurs in non-Indigenous people, the increase is less pronounced.

**Table 2:** Total life expectancy, disability-free life expectancy and disabled life expectancy among Indigenous (Mapuche) and non-Indigenous older adults in Chile

Ethnic Origin	TLE	DFLE (CI 95%)	DLE (CI 95%)	Ratio DLE/DFLE
**Mapuche**				
60 years	18.9	9.4 (8.4–10.3)	9.5 (8.6–10.5)	1.0
70 years	11.0	4.3 (3.6-5.0)	6.6 (5.9–7.3)	1.5
80 years	5.3	0.8 (0.4–1.2)	4.4 (4.0-4.8)	5.3
90 + years	3.6	0.3 (0.0-0.9)	3.3 (2.8–3.9)	11.0
**Non-Indigenous**				
60 years	26.4	14.0 (12.8–15.3)	12.3 (11.1–13.6)	0.9
70 years	17.3	8.0 (6.9–9.1)	9.3 (8.2–10.5)	1.2
80 years	9.6	3.6 (2.5–4.6)	6.0 (5.0-7.1)	1.7
90 + years	4.5	1.1 (0.0-2.2)	3.4 (2.3–4.5)	3.0

*Source*: Own elaboration using EDES Survey and Life Table by Ethnicity [31]

TLE: Total life expectancy, DFLE: Disability-free life expectancy, DLE: Disability life expectancy

**Fig. 2:**
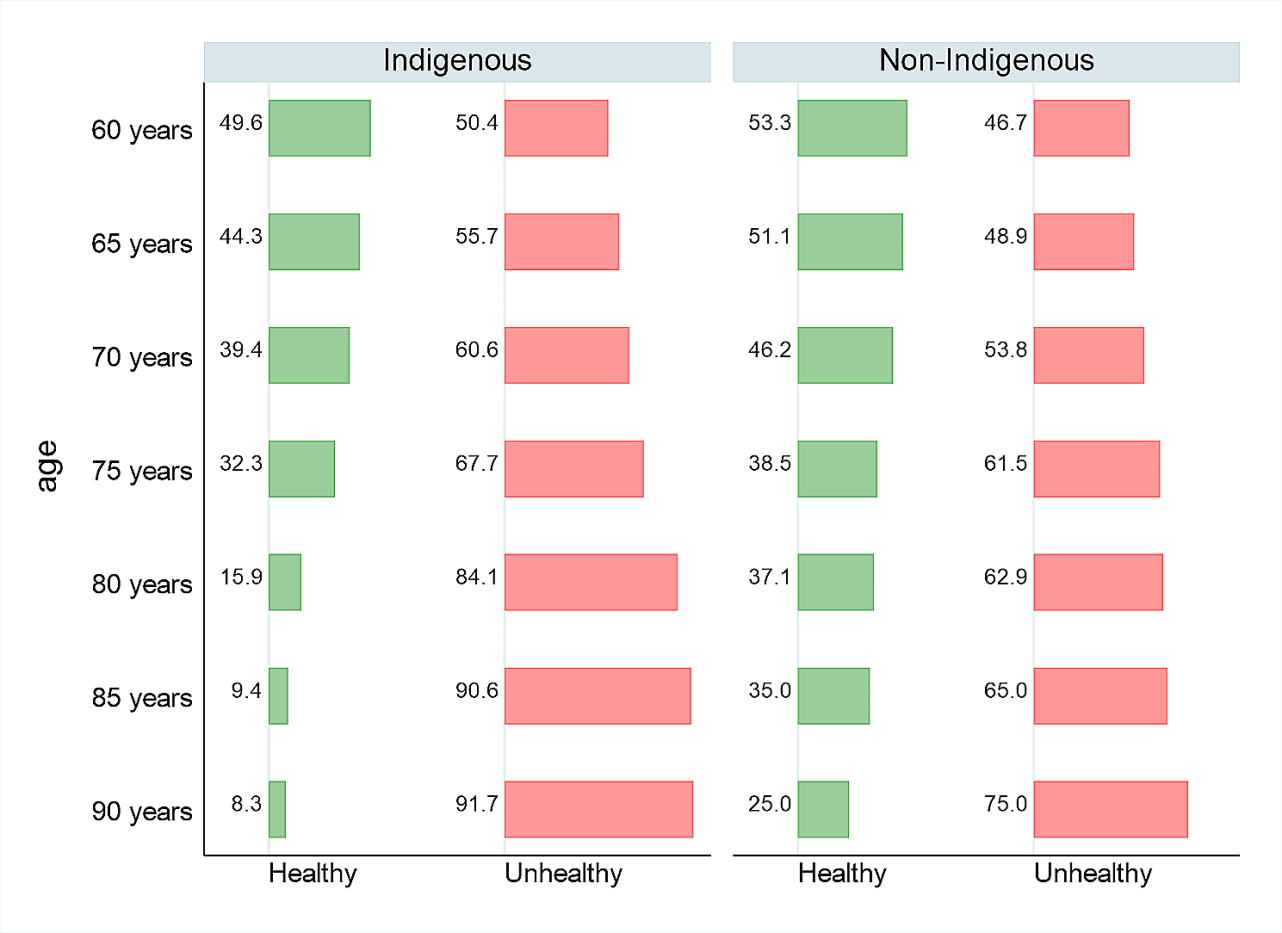
Proportion of life spent disability-free (Healthy) and proportion with disability (Unhealthy) among Indigenous (Mapuche) and non-Indigenous older adults in Chile. *Source*: Own Elaboration using EDES Survey and Life Table by Ethnicity [31]

This is shown more clearly in Fig. 2, which indicates that older Mapuches have a larger proportion of disabled life expectancy than non-Indigenous persons at all ages. This increases considerably at age 80 and above; although an 80-year-old Mapuche may expect to live 5.3 years (TLE), only 15.9% can expect to do so free of disability. This is less than half (15.9% vs. 37.1%) the proportion for non-Indigenous older adults. The figure also indicates that 91% of the Mapuches who live to age 90 can expect to be unhealthy (with disability).

## Discussion

Our results confirm that Mapuche older adults in Chile have a double disadvantage in survival compared to non-Indigenous persons, because they live fewer years, and live a larger proportion of these years with disability (unhealthy state) than do non-Indigenous people. The magnitude of this ethnic difference in disability-free life expectancy was previously unknown.

This study is in line with results in other countries that have shown ethnic-racial differences in longevity. This is the case of the United States of America (USA), a country in which ethnic-racial differentials in total, healthy (or disability-free) life expectancy has perhaps been studied in greater detail [56–58].. For example, Hayward and Heron [58] found that for adults (20–85 years) people of African descent live few years and they live a high proportion of those years with chronic health problems. Meanwhile, Solé-Auró et al. [57] analyzing disability-free life expectancy between 60 and 90 years of age according to gender, race, and educational level, detected that disability-free life expectancy only increased among white men, whereas life expectancy with disability increased in all groups: white and black men and women. Recently Dwyer-Lindgren et al. [59] found that differences in life expectancy between racial/ethnic groups (comparing non-Latino White, non-Latino Black, non-Latino American Indian and Alaska Native, non-Latino Asian and Pacific Islander, and Latino or Hispanic) have widened and hardened in the period 2000 and 2019. Although our focus of study has been the comparison between the largest group of indigenous Chileans (Mapuche) with the non-indigenous (who could be the simile of Latinos or Hispanics in the USA), the results are consistent with the evidence described for that more industrialized society: ethnic minorities, in our case, indigenous Mapuche, see their chances of having a healthy and prolonged life truncated.

It has also been shown that Indigenous persons have poorer health results compared to non-Indigenous people in almost all regions of the world [3, 33, 60, 61]. For Australia and New Zealand, for example, it was reported that Indigenous people expect to live 13–20 fewer years than non-Indigenous persons [62–64]. A similar disadvantage for Indigenous persons has been documented in Latin America, but of lower magnitude [31, 34, 38, 65–67]. For example, in a previous study [31] we found that the life expectancy at birth for Indigenous people is 7 years less than non-Indigenous persons, which at age 60 reduces to six years. However, according to our review, this is the first Latin American study focused on comparing the disability and disability-free life expectancy between Indigenous (Mapuche in our case) and non-Indigenous older adults.

Although the economic growth of Chile has made it one of the countries with high income (Gross Domestic Product = $30,266 per capita) [68], it does not differ from other Latin American countries in the structural inequalities that affect its population, especially Indigenous people. The resources and living conditions associated with low socioeconomic status are a fundamental cause of health inequalities [69]. The existing evidence is compelling: people with low socioeconomic status have high rates of functional decline, experience shorter life spans, and experience a greater number of years in poor health [6, 8, 70]. The reality of the indigenous population detected in this study - shorter LE and DLFE - we believe that it is highly associated with the socioeconomic disadvantages that affect them. For example, in 2017–2020 the poverty rate of Indigenous people increased from 15.4 to 17.1%, which are relatively higher than that for non-Indigenous people (10.2–13.9% for the same period) [71]. The ethnic inequalities in health indicators in Chile are persistent, including child and adult mortality. For example, Amigo and Bustos [72] used national data from 2000 to 2004 and distinguished children with none, one or two Indigenous surnames (two surnames are used in Chile), found that child mortality for those with two Indigenous surnames was 17.1 per 1000 births, while for non-Indigenous child it was 8.8. Similarly, CEPAL [33] reported that in the 2010-decade Indigenous children had a mortality rate 11% greater than non-Indigenous. Indigenous people also have greater chances of suffering chronic diseases like diabetes [73] and mental illnesses [74, 75]. Knowing that there is a wide literature associating poverty with poorer health, and that the Indigenous population has poorer health, it is not surprising that Mapuche have a lower disability-free life expectancy than non-Indigenous Chileans.

The results shown in this study highlight the need to deepen the implementation of socioeconomic and health policies that really contribute to eradicate the disadvantages in terms of disability and mortality that greatly affect the Mapuche in comparison with non-indigenous people. Health promotion and care policies that consider an intercultural perspective and a life course perspective can be a good tool for the promotion of healthy habits and an active life in the Chilean population, especially among the indigenous population. Our study has some limitations. The first is the gap between the main information sources. The estimation of the life table by ethnic origin used data from the 2017 census, while the prevalence of disability came from EDES 2022; this could produce an unmeasured bias. Although this is not a minor limitation, the sources used in this work are the only ones existing to date that allow us to approach the phenomenon of ethnic differentials in disability-free life expectancy in Chile. Second, since there is no information available to estimate life tables by sex and ethnic origin, our results are for both sexes together. Thus, we cannot speak to the discussion about differences in disability-free life expectancy between sexes or on the intersection between gender and ethnicity. Third, the disability prevalence data come from a representative sample of older Mapuche and non-Indigenous adults of the two regions of the country with the highest proportions of Indigenous people. Although it is probable that disability prevalence varies among Indigenous peoples, it was not possible to examine this in this study. Despite these limitations, the study has important strengths: the results are the first attempt to demonstrate ethnic differences in the number and quality of years of life of older adults and therefore are of great relevance for Chile and Latin America. This is an important aspect for the design and implementation of public policies tending to decrease the breach in longevity detected here. Future studies should explore the effects of gender and ethnic origin on longevity.

## Conclusions

The results of our study provide a completely unknown picture in the country. Although they are in line with what was expected - indigenous people expect to live shorter and healthier lives than non-indigenous older adults - given that population-level changes in health and longevity tend to occur slowly, longitudinal information is needed in the future to investigate such changes considering ethnic-racial differences.

In line with the National Health Strategy for the Sanitary Objectives of 2030 [76], which established among its objectives to “improve the health and wellbeing of the population” and “decrease the inequalities”, we believe that the results of this study should be the basis for the development of public policies oriented to reduce the ethnic differences in health and longevity of the Chilean population. Our results also underscore the need for public institutions to increase their capacity to monitor mortality risks of older persons, considering ethnic differences. It is highly desirable for Chile to compile systematically information about ethnic origin in death certificates [35]. This information would undoubtedly favor a more complete and inclusive analysis of the inequalities in longevity and mortality among older adults of different ethnic-racial origins.

## Data Availability

The datasets generated and/or analyzed during the current study are not publicly available due to restrictions associated with data ownership but are available from the corresponding author on reasonable request.
